# Barriers in motion: a multi-methods approach to exploring physical activity, physical function, and program challenges in adult day services

**DOI:** 10.3389/fpubh.2025.1620038

**Published:** 2025-10-24

**Authors:** Yuliana Soto, Jacqueline Guzman, Diana Morales, Imani Canton, Ana Selzer, Susan Aguinaga

**Affiliations:** ^1^Exercise Psychology Lab, Department of Medicine, University of Illinois at Chicago, Chicago, IL, United States; ^2^Cancer Center, Medical College of Wisconsin, Milwaukee, WI, United States; ^3^Health Equity and Aging Lab, Department of Health and Kinesiology, University of Illinois at Urbana-Champaign, Urbana, IL, United States; ^4^Yale School of Public Health, Yale University, New Haven, CT, United States

**Keywords:** long-term care services, aging in place, older adults, health equity, community-based care

## Abstract

**Introduction:**

Physical activity (PA) helps older adults age in place and retain independence. Adult Day Centers (ADCs) are critical community-based spaces that provide PA programming, yet the dosage and impact of PA in these settings remains empirically unassessed.

**Methods:**

This study used a multi-methods cross-sectional design to assess PA and physical function among ADC participants, as well as directors’ perspectives on PA programming. PA was assessed via an ActivPAL inclinometer, and physical function was assessed via the Short Physical Performance Battery (SPPB) and the Timed Up and Go (TUG) test. Semi-structured interviews were conducted with ADC directors. Data from interviews was coded openly and axially, and analyzed inductively and deductively to extract major themes. The qualitative analysis was subsequently guided by the Frequency, Intensity, Type, and Time principles to assess PA dosage.

**Results:**

On average, ADC participants (*N* = 48; M_Age_ = 74.8 ± 7.2; 78.6% Female; 76.9% Low-Income; 70.7% Hispanic) engaged in 36.4 ± 28.8 min of moderate to vigorous PA per day, with 68% of participants meeting the PA guidelines. Physical function scores indicated an elevated risk for falls, morbidity, and mortality (M_SPPB_ = 8.8 ± 2.1, M_TUG_ = 14.7 ± 4.0). Interviews with five ADC directors revealed overarching themes: (1) PA dosage and programming at ADCs, (2) barriers to PA (staff shortages, funding, and safety and liability), and (3) programming facilitators.

**Discussion:**

Findings reflect broader systemic challenges that influence PA programming at ADCs. The directors cited barriers such as staffing limitations, funding constraints, and safety concerns, emphasizing the need for and desire to receive additional support. These challenges were also reflected in the ADC participants’ PA and physical function. It is imperative to support ADCs in delivering evidence-based programming as they can be key to retaining physical functional status and improving the quality of life of ADC participants. Future studies should consider community-based strategies involving liaisons and PA experts to support ADC staff, increase PA training, and reduce staff burden and turnover.

## 1 Introduction

Adult day center (ADC) participants represent a segment of the older adult population with greater health and functional challenges than their community-dwelling peers (1, 2). Many ADC participants live with multiple chronic conditions, cognitive decline, and require daily supervision, which contributes to lower levels of physical activity (PA) and higher levels of sedentary behavior (3, 4), placing them at elevated risk for cardiovascular disease, dementia, diabetes, falls, and functional decline (5). Given these vulnerabilities, ADCs are a critical setting for examining PA patterns and identifying strategies to preserve independence and quality of life in this population.

ADCs are community-based facilities, staffed by nurses, social workers, and aides that provide long-term care services to support the health, medical, social, nutritional, and activity needs of older adults (2). These centers offer a structured and supervised environment that makes them well-positioned for promoting PA and preserving physical functioning. ADCs provide a safe space, staff supervision, the ability to embed structured daily PA programming, and direct contact with caregivers to provide them with recommendations (6). In 2018, across the United States, there were approximately 251,000 ADC participants, most of which were female, had 2–3 chronic conditions such as high blood pressure and diabetes, and were Medicaid beneficiaries (9). In the 2024 fiscal year, the Illinois Department of Aging reported that 74 ADCs held contracts to deliver community-based care, serving an average of 1,300 older adults each month across the state (7).

These facilities are becoming a preferred option compared to traditional nursing home facilities for community-based long-term care for older adults with chronic health conditions, as they support families and caregivers while enabling participants to age in place (8, 9). ADCs serve as critical resources for immigrants and marginalized populations, who may prefer ADCs due to their alignment with cultural values such as familism and collectivism (10, 11). Given the elevated risk of chronic disease, Alzheimer’s disease, disability, and poor quality of life among marginalized populations, ADCs represent a vital setting for the promotion of healthy behaviors, including PA.

PA is a low-cost, accessible, and non-pharmacological strategy for addressing health disparities and promoting aging in place among vulnerable populations (12). Studies show that aging in place is beneficial for most older adults, and it is more likely to be achieved or maintained when supportive environmental conditions are present (13). Thus, promoting PA participation in ADCs represents a meaningful public health opportunity, especially for managing and preventing chronic diseases, and maintaining the functional capacity needed for independent living and facilitating aging in place.

While many ADCs report offering PA programs, the amount or type of PA is rarely described, nor is PA a required or mandated service. Furthermore, there is limited empirical evidence on device-assessed PA and physical functioning among ADC participants. Data on ADCs report on physical therapy services (6), but critical details regarding the quantity and quality of PA, as well as key elements of PA prescription are lacking. The FITT principle, which outlines frequency, intensity, time, and type of PA necessary to achieve health benefits, may provide a framework for assessing PA dosage in these settings (14). Determining whether ADC participants meet national PA guidelines is essential, as national guidelines recommend at least 150 min of moderate-to-vigorous physical activity (MVPA) per week to promote health and prevent functional decline (15).

Assessing PA within ADCs will provide a more comprehensive understanding of their role in supporting the health and well-being of older adults. Additionally, evaluating the PA of participants while at an ADC, as well as the perspectives of ADC directors on PA programming, can provide insight into the barriers and opportunities for improving PA programming in these settings. The present study used a multi-methods approach to assess the PA and physical function levels of ADC participants, as well as the ADC director’s perspectives on PA programming. Given current low levels of PA participation among older adults, we hypothesize that ADC participants will not meet the PA guidelines and that their physical function scores will be indicative of impairment. We further hypothesize variability across centers, reflecting differences in PA programming.

## 2 Materials and methods

### 2.1 Design

The current study used a cross-sectional multi-methods design to assess the PA and physical function of ADC participants and understand the perspectives of ADC directors on PA programming at their respective centers. The purpose of employing a multi-methods approach was to complement findings from different methodologies while examining related phenomena. Quantitative and qualitative data were analyzed separately and integrated sequentially during interpretation to provide a more comprehensive understanding of PA engagement and program delivery in ADCs.

### 2.2 Sample

Data from ADC participants were obtained from two studies, STAND-UP and LUCID, and were included in the present analysis.

#### 2.2.1 STAND-UP

STAND-UP was a prospective study in ADCs to examine PA, physical function, and psychological well-being of participants. Twenty-seven participants were recruited from five ADCs in Chicago, the surrounding suburbs, and central Illinois. ADC participants with racially and ethnically diverse backgrounds were prioritized; however, non-Hispanic White participants were not excluded. STAND-UP was approved by the IRB BLINDED FOR REVIEW.

Inclusion criteria for participants included attending the ADC, age ≥ 60 years, being able to understand Spanish or English, and having a Mini-Mental State Exam score ≥ 18. Participants were excluded if they had self-reported significant physical illness, medical condition, or current/past history of a significant psychiatric condition that would interfere with participation in the study, could not walk 15 feet without regular use of an assistive device (e.g., cane, walker), had a Mini-Mental State Exam score <18 (e.g., indicating moderate to severe cognitive decline).

#### 2.2.2 LUCID

LUCID was an intervention study evaluating the effects of a Latin Dance program on cognition and PA among older Latinos with mild impairment. Baseline PA data from *n* = 21 ADC participants who previously participated LUCID, were combined with the above-described sample. Detailed information on participants’ characteristics and recruitment can be found elsewhere (BLINDED FOR REVIEW). Participants were Spanish-speaking older Latinos (75.4 ± 6.3 years old, 16 females and 5 males, with a Mini-Mental State Examination score of 22.4 ± 2.8). LUCID was approved by the IRB BLINDED FOR REVIEW.

Inclusion criteria for participation were being at least 60 years old, identifying as Latino or Hispanic, speaking or understanding Spanish, and scoring between 18 and 26 on the MMSE, indicative of mild cognitive impairment. Exclusion criteria included regular use of a mobility aid (e.g., cane), a history of stroke, or self-reporting more than 150 min per week of structured aerobic exercise.

### 2.3 Recruitment procedures

ADC participants were recruited from ADCs through flyers and announcements. ADC directors were asked via face-to-face interaction to participate in interviews to discuss the implementation of PA programming at their respective centers. Eligibility criteria for directors included serving in a leadership capacity within the ADC and being able to answer questions regarding PA programming at their respective centers. ADC directors were recruited exclusively from the same facilities where participant data were collected to ensure that the qualitative findings directly reflected the organizational context in which participants’ PA was assessed.

### 2.4 Data collection

Data were collected from 2016 to 2019. All participants signed informed consent in their preferred language. All procedures were done in a private room at the participants’ respective ADCs. Directors provided consent during their interview day. All study procedures were approved by the Institutional Review Board of BLINDED FOR REVIEW.

### 2.5 Measures

#### 2.5.1 ADC participants

Demographic data included age, race, ethnicity, education, and income. Height and weight were measured via a stadiometer and a digital scale to calculate body mass index (BMI; kg/m^2^). Cognitive function was assessed using the Mini-Mental State Exam (MMSE), a widely used clinical screening tool designed to evaluate the cognitive status of older adults. The MMSE assesses various domains of cognition, including orientation to time and place, attention and calculation, immediate and short-term memory, language ability, and visuospatial abilities. The test scores range from 0 to 30 points, with scores below 27 indicating cognitive decline (16).

Physical function was assessed via the Short Physical Performance Battery (SPPB), an objective, validated assessment tool for evaluating lower extremity functioning in older adults (17). The SPPB consists of a series of physical performance tests, including balance tests (e.g., side by side, semi-tandem, full tandem), 3-meter gait speed tests, and chair stands. SPPB scores range from 0 to 12, with higher scores indicating greater lower extremity function. Participants wore a gait belt for safety precautions. Participants also completed the Timed Up and Go (TUG). The TUG test is a reliable, cost-effective, safe way to evaluate overall functional mobility. The TUG involves participants rising from an armless chair (46 cm in height), walking 3 m at a normal pace and turning around on a marked floor, walking back, and sitting again (18). Time was recorded when participants’ buttocks were lifted off the chair to stand and ceased when the buttocks touched the seat when returning to a sitting position. TUG has excellent intra-rater reliability in community-dwelling older adults (19) and moderate to excellent validity in older adults with and without cognitive impairment (20).

PA was assessed via an ActivPAL 3TM® inclinometer monitor (PAL Technologies, Glasgow, Scotland, the United Kingdom) worn on the thigh. Participants were instructed to keep the monitor on for seven continuous days. Each individual received written placement instructions and additional adhesive dressings in the event that they needed to remove the monitor and reattach it. They were also asked to complete a sleep log to record wake time and sleep time. The “Events” files were processed and extracted from ActivPal3 software to analyze data; these list all bouts of sitting/lying, standing, and steps, with the time each bout begins and duration (21). Valid wear-time was defined as a minimum of 10 h/day, with at least 3 valid days required for inclusion. Non-wear time was identified as ≥60 consecutive minutes of zero counts, consistent with standard protocols. Given that most did not submit a sleep log, data was visually inspected to isolate waking time by visually identifying sleep times. If extended bouts of sitting/lying were identified around late evening or early morning, those segments would be removed from the file (21). Activity was classified as sedentary based on the sum of time where the activity code was 0, standing as the sum of time where the activity code was 1, light activity as the sum of time where the activity code was 1 or 2 and METs were less than or equal to 3, and MVPA as sum of time where METs are > 3. The percentage of participants meeting PA guidelines (≥150 min of MVPA per week) was calculated by multiplying each participant’s average daily MVPA by 7 days. Participants meeting or exceeding this threshold were coded as meeting guidelines.

#### 2.5.2 Qualitative interviews with ADC directors

Directors interested in participating in the study were debriefed on the study components. After providing consent, participants took part in a semi-structured, in-person interview of approximately 30 min to assess their perceptions of barriers and facilitators to exercise in an ADC setting. All interviews were conducted by the study principal investigator (SA), who has extensive experience conducting community-based research with older adults. The PI has training in semi-structured interviewing and followed a structured interview guide (see Table 1) to ensure consistency across interviews.

**Table 1:** Semi-structured interview guide for adult day center directors.

Domain/topic	Example questions
Program structure	Can you describe the physical activity (PA) programs currently offered at your center? How often are these activities scheduled?
Types of activities	What types of PA do participants usually engage in? Do you use any structured or evidence-based PA programs (e.g., yoga, aerobics, strength training)?
Intensity and duration	How long do sessions typically last? How would you describe the intensity of the activities (light, moderate, vigorous)?
Staff roles and training	Who typically leads the PA sessions? Have staff received any specific training or certification for leading PA activities?
Barriers and challenges	What challenges have you encountered when implementing PA programs? How do staffing, funding, or liability concerns affect your ability to deliver PA programming?
Facilitators and resources	What resources or supports (internal or external) help you deliver PA programs? Do you collaborate with community partners or use volunteers?
Participant engagement	How do participants respond to the PA activities offered? Do you receive feedback from participants or caregivers on the PA programming?
Future opportunities	What changes or improvements would you like to see in PA programming at your center? What support would make PA programming more sustainable?

### 2.6 Statistical analyses

#### 2.6.1 Quantitative

All descriptive statistical analyses were performed using SPSS software, version 27 (IBM Corp., NY, USA). The percentage of missing data was 33.3% for PA outcomes (16/48 cases), 12.5% for SPPB (6/48 cases), and 4.35% for TUG (2/46 cases). Due to the presence of missing data that was determined not to be missing completely at random (MCAR) (Little’s MCAR test = *χ*^2^ (6) = 16.77, *p* = 0.01). Missing PA data (33.3% of participants) were primarily due to lost devices, with a smaller proportion attributable to non-compliance or technical issues. Given that many participants demonstrated mild cognitive impairment, cognitive limitations may also have contributed to device loss or difficulties with compliance. Multiple imputation was conducted to address missing data for SPPB, TUG, and PA. Five imputations were performed using fully conditional specification (FCS) in SPSS, which estimates missing values based on the relationship among observed data, including all available demographic and outcome variables as predictors in the model. Given the descriptive nature of the analyses and absence of sensitivity checks, findings should be interpreted cautiously.

#### 2.6.2 Qualitative

Director interviews were semi-structured, audiotaped, and transcribed verbatim by a research staff member. Data from two interview transcripts were open and axially coded through an inductive and deductive approach (22). A deductive approach was used, with initial codes obtained from the FITT principle (Frequency, Intensity, Time, and Type). Interview excerpts describing PA programming were mapped onto these dimensions; for example, references to how often activities were offered were coded under Frequency, descriptions of exertion levels were coded under Intensity, reported session lengths were coded under Time, and types of activities (e.g., dance, walking, chair exercises) were coded under Type.

Open and axial coding was done to examine links to other codes and to group codes that might represent a common theme (23). Constant comparison and contrasting within and across different transcripts (24) were done to examine similarities and differences within the data. Three members of the research team (JG, IC, AS) independently coded transcripts, and consistency was achieved through consensus meetings. Codes were discussed by the research team to design a preliminary codebook. Once the research team reviewed the preliminary codebook, the codebook was used to analyze the remaining transcripts. After coding all the remaining interviews, the research team met to discuss the agreement on the final codebook and recoded the initial transcripts to ensure the consistency of the codebook. Because the study included all available directors (*n* = 5), thematic saturation was not expected; however, the codebook was applied across all transcripts to ensure completeness and consistency.

## 3 Results

The study included a total of 48 ADC participants across six ADCs, with 70% self-identifying as Latino/Hispanic and 78.6% female. Participants had a mean age of 74.8 ± 7.2 years, an MMSE score of 24.1 ± 3.4, indicating mild cognitive impairment, and 76.9% of the sample reported an income below $25,000, reflecting a sample with low socioeconomic status. Participants had an average of 9.68 ± 6.46 years of formal education (Table 2).

**Table 2:** Demographics of adult day center participants.

Characteristics	*N*	Mean (SD) or %
Age	48	74.75 (±7.19)
Female	33	78.6%
BMI	47	28.39 (±6.30)
Ethnicity	41	
Hispanic or Latino	29	70.7%
Not Hispanic or Latino	12	29.3%
Race	34	
African American	13	38.2%
White	9	26.5%
Native American	2	5.9%
Mixed	2	5.9%
Unknown	8	23.5%
Home income	39	
< $25,000	30	76.9%
≥ $25,000	3	7.6%
Unknown	6	15.4%
Years of education	32	
0–9 years	20	62.5%
10–18 years	8	25%
18 +	4	12.5%
MMSE	48	24.06 (±3.38)
SPPB Score	42	8.79 (±2.10)
TUG (s)	42	14.69 (±4.04)
Minutes of MVPA/day	32	36.36 (±28.83)
Sedentary hours/day		14.49 (±2.60)
Light Activity hours/day		5.02 (±2.88)
Standing hours/day		4.50 (±2.64)
ADC # 1	5	8.6%
ADC # 2	6	10.3%
ADC # 3	21	36.2%
ADC # 4	5	8.6%
ADC # 5	1	1.7%
ADC # 6	10	17.2%

### 3.1 Physical function of ADC participants

On average, participants were at high risk of physical limitations with SPPB scores of 8.8 ± 2.1. A score of less than 10 on SPPB indicates the participant has one or more mobility limitations and is predictive of all-cause mortality (17). In the TUG test, participants scored an average of 14.7 ± 4.0 s; a score of 14 s or more indicates high risk for falls (25).

### 3.2 Device-assessed PA of ADC participants

**Figure 1:**
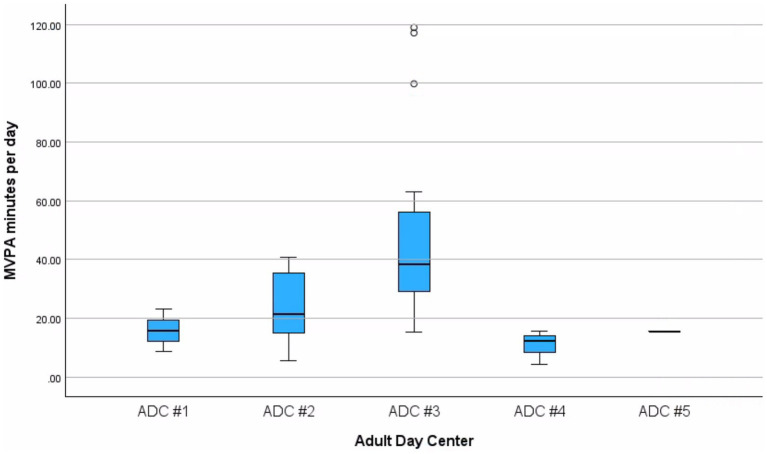
Distribution of daily MVPA minutes across adult day centers. Box plots display medians, interquartile ranges (IQR), and minimum/maximum values, with circles representing outliers (>1.5 × IQR). Variation across centers should be interpreted descriptively, as uneven sample sizes (range *n* = 1–21) limit inferential comparisons.

Overall, 68.75% of participants met the recommended 150 min of MVPA per week, while 31.25% did not. PA levels among participants varied across centers. On average, ADC participants engaged in 5.02 ± 2.88 h of light PA per day, 36.36 ± 28.83 min of MVPA per day, and 14.49 ± 2.60 of sedentary hours per day. The most active center was ADC # 3 and averaged 48.87 ± 30.82 min of MVPA per day, while the least active center, ADC # 4, engaged in 10.8 ± 5.75 min of MVPA per day. Figure 1 illustrates the distribution of MVPA minutes per day across centers, highlighting variability in activity levels.

Exploratory correlations indicated that MVPA was positively associated with physical function (SPPB: *r* = 0.28, Chair Stand: *r* = 0.46, Gait: *r* = 0.33; all *p* < 0.001) but negatively correlated with MMSE (*r* = −0.48, *p* < 0.001). Additionally, MVPA was modestly lower among women (*r* = −0.32, *p* < 0.001). No associations were observed with age. While cognitive functioning was positively associated with income (*r* = 0.25, *p* < 0.01).

### 3.3 Qualitative interviews

Five ADC directors participated in interviews with an average interview length of 21:06 ± 10:00 min. Two major themes were extracted: (1) PA programming at ADCs through a FITT principle lens and (2) barriers to PA implementation. Directors detailed PA activity patterns, including frequency, intensity, time, and type (FITT), and identified facilitators and three key barriers: staff training, funding constraints, and safety/liability concerns.

#### 3.3.1 PA programming at ADCs: a FITT principle lens

Four out of five directors reported offering daily PA programming, while one center reported it once a week, as shown in Table 3. Directors described most activities as low-intensity, light stretches, and soft movements. Only one director at ADC # 4 reported moderate-intensity aerobic exercise. The duration and frequency of PA varied across centers from a few minutes a day to 45 min per day and from once a week to multiple times a day. The most common activities reported were chair-based exercises, seated yoga, and dance. A few centers indicated using exercise drums, elastic bands, small-pound weights, and waving balloons or scarves in the air.

**Table 3:** Physical activities offered at adult day centers based on the FITT principle and average of physical activity per day.

ADC	Frequency	Intensity	Time	Type	Average device-assessed physical activity per day (SD)
ADC # 1 Non-Profit	Everyday	Not too strenuous or too aerobic	30 min	Aerobic: Dancing, sports (e.g., volleyball), walking (e.g., field trips) Resistance: Sitting exercises (e.g., lifting legs and arms)	Minutes of MVPA: 15.94 (7.20) Sedentary hours: 12.44 (1.41) Light activity hours: 2.60 (1.39) Standing hours: 2.26 (1.20)
ADC # 2 Non-Profit	Everyday	Soft movement, no jumping	20–30 min	Aerobic: Dancing (e.g., Hokey Pokey), walking, sitting exercises (e.g., dancing in seat) Flexibility: Overall stretching exercises	Minutes of MVPA: 23.26 (13.06) Sedentary hours: 9.64 (2.69) Light activity hours: 4.59 (2.12) Standing hours: 4.23 (2.11)
ADC # 3 Non-Profit	Everyday	Low impact aerobics	Multiple times throughout the day	Aerobic: Walking, dancing, pedaling classes Resistance: Small pound weights, elastic bands, slow dancing, pedals Flexibility: Stretching exercises, yoga	Minutes of MVPA: 48.87 (30.82) Sedentary hours: 11.02 (2.52) Light activity hours: 6.24 (2.76) Standing hours: 5.58 (2.53)
ADC # 4 Private	Everyday	Moderate to get heart rate up	1.5 h	Aerobic: Sitting exercises (arm and leg movements, dancing) Resistance: One pound weight exercises Flexibility: Stretching of hands	Minutes of MVPA: 10.80 (5.75) Sedentary hours: 7.65 (1.71) Light activity hours: 1.80 (1.13) Standing hours: 1.56 (1.02)
ADC # 5 Non-Profit	Once a week	Light stretches/muscle toning	Varies, few minutes	Aerobic: Dancing, Tai chi Resistance: Sitting exercises, elastic bands, yoga and Tai chi Flexibility: Yoga	Minutes of MVPA: 15.52 Sedentary hours: 8.21 Light activity hours: 1.49 Standing hours: 1.27

ADC # 6 Director did not participate in interviews at baseline. Sample sizes varied substantially across centers (range *n* = 1–21), limiting the interpretability of center-level comparisons. Data are descriptive only.

#### 3.3.2 Barriers to PA programming at ADCs

Several barriers to PA programming emerged from the director interviews, with three primary subthemes identified: staff shortages, funding limitations, and safety and liability concerns. However, a deeper analysis reveals that these barriers are interconnected, with staff training and high turnover serving as an underlying theme that influences and exacerbates each of these challenges.

##### 3.3.2.1 Staff shortages

A common challenge with implementing exercise programs and expanding the types of exercises ADCs can offer is the shortage of staff. Although center directors were not explicitly asked about their staff members, all centers highlighted difficulties in delivering exercise programs due to limited staff. For example, when asked about using external resources or infrastructure for exercise, such as public parks, pools, or other community centers, directors explained that these options were not feasible because they would require multiple staff members to assist the participants.

I don’t know. I mean it’ll take a lot of staff time […] If we had two people [participants], we would have to have two staff because getting them changed into their swimming suits and monitoring them […] Getting them safely in and out of the pool and not slip and dressed that is […] one to one.

*(ADC # 4)*

Center directors also noted that many ADC participants have specific needs requiring special attention, such as mobility limitations or dementia. The number of staff determines the activities they can offer at the center. A center director mentions, “We have some that cannot move, have to be fed, so we would not be able to [offer certain activities] here because of our limitations” (ADC # 1). This challenge is exacerbated by constant staff turnover and student schedules. Some ADCs with established partnerships with universities rely on student interns to lead PA programming. However, when volunteer instructors or student interns are unavailable, the responsibility falls again on center staff, who must juggle PA programming alongside other duties. As one director explained, “We used to have a Tai chi person come in… and also a physical [therapy] person come in once a month to do Zumba with them… but that was before I was hired. I think it was just a volunteer who no longer comes in” (ADC #5). This highlights how centers rely on external support to expand PA offerings, support that can be inconsistent or short-lived.

##### 3.3.2.2 Funding

While directors did not directly identify funding as a barrier to exercise programming, it played a significant role in the types of exercises ADCs can offer. When specifically asked about funding for PA programming delivery, all directors stated that funding was not an issue. However, they did acknowledge funding as a barrier to hiring exercise professionals or providing staff certifications needed to deliver PA programming effectively. As one director explained, “Our budget is pretty slim, so we try to tailor things… such as going on field trips, we try to go towards the free things… because, you know, the budget is kind of tight with us being a non-profit organization” (ADC # 5).

Staff are required to complete 8–12 h of annual training, and while some centers manage to certify staff to lead PA programming, tight budgets and high turnover make this difficult for others. Directors noted it’s hard to justify training costs when staff may leave shortly after certification.

They [community resources] charge for training for two or three days, and then I see who I can send. That is also risky because one can pay to train a staff member, and then they leave. But we try to send someone to get the certificate and then have them give classes here… and then they need to get recertified every year or two.

*(ADC # 3)*

##### 3.3.2.3 Safety and liability

Different concerns emerged in relation to the safety of participants while participating in PA programming. The main concern across all directors was the risk of falls and the associated liability. For example, two out of five directors expressed concerns about falls during walks in the park due to uneven pavement.

I have the park here in front. We cannot walk around because we have already tried, and if they fall… it is not easy to see the floor crack, a little thing, so they fall down. So we stopped doing that. […] Then we risk that someone falls and breaks an arm then it is a problem for [the] ADC.

*(ADC # 3)*

Directors also expressed concerns about the types of activities and whether they required standing, balance, or mobility. A director reported feeling uncomfortable with the participants engaging in certain types of PA, “Everyone is so fearful of falling, Tai chi is standing up, you know […] That would be something I would be uncomfortable with” (ADC # 4). Another director noted that cognitive impairment posed additional constraints, “over 80% of my participants have dementia, so we are restricted from things like pool visits. Safety is a concern if we go out” (ADC #1). The fear of participant falls and the associated liability concerns led directors to adapt activities to ensure the safety of both their participants and organizations. As a result, all ADCs prioritized seated or chair-based exercises, driven by concerns over fall risks. While the ADCs want to provide exercise opportunities for their participants, it is also crucial that they deliver it in a safe manner.

The staff have already agreed on the movements. Simply stretch, stretch your back, arms, legs, and monitoring. That there is no one doing something that should not or will lose balance and fall. Everything that we are doing. As long as we keep them active, moving, and not sitting for many hours.

*(ADC # 3)*

#### 3.3.3 Facilitators of PA programming at ADCs

Although directors emphasized barriers strongly, several supportive elements also emerged. A recurring facilitator was the adaptation of activities to participant abilities, such as seated or chair-based exercises, which enabled greater participation among individuals with mobility or cognitive impairments. One director explained, “We do chair exercises so everyone can join in, even those who cannot stand for long” (ADC #1). Directors also described embedding active games into daily schedules to keep participants engaged. In some cases, ADCs benefited from staff, volunteers, or student interns who could lead activities, as well as collaborations with outside organizations that provided additional PA opportunities. Directors expressed interest in potential collaborations with community partners and highlighted that PA often fostered socialization, which motivated participation: “As long as we keep them active, moving, and not sitting for many hours” (ADC #3). Together, these facilitators highlight that even within constrained settings, adaptation, social engagement, and external support play important roles in enabling PA.

## 4 Discussion

This study aimed to describe PA levels among ADC participants using a multi-methods approach, integrating device-assessed PA data, physical function measures, and qualitative interviews with ADC directors. Findings indicate that about a third of ADC participants did not meet recommended PA guidelines, engaged in high levels of sedentary behavior, and demonstrated functional limitations that increased their risk for falls. While directors reported offering PA programs, the frequency, duration, and intensity of activities varied considerably, with most falling below recommended intensity levels. Directors also identified systemic challenges, including staffing shortages, inconsistent training, and liability concerns, all of which limit older adults’ opportunities to engage in meaningful PA.

PA levels varied significantly across ADCs. The U.S. Department of Health and Human Services (15) and the World Health Organization (26) recommend at least 150 min of MVPA per week. In our sample, 68.75% of participants met this threshold, while nearly one-third fell below guidelines. Thus, our hypothesis that most participants would not meet PA guidelines was only partially supported. Notably, participants at ADC # 4 engaged in an average of 10.8 min of MVPA per day, compared to 48.87 min per day at ADC # 3. Interviews provided contextual insight into this disparity, with ADC # 3 offering daily PA programming, including walking and aerobics, while ADC #4 primarily reported seated exercises. Additionally, participants in our study had a mean of 14.49 ± 2.60 h per day of sedentary time, a concerning figure given its association with sarcopenia, mobility decline, increased fall risk (27), and overall mortality risk (28). To our knowledge, this is one of the first studies to employ device-assessed PA data across several ADCs. Without structured, progressive PA interventions, ADC participants face heightened risks of mobility decline, functional dependence, and diminished quality of life (29). Integration of our quantitative and qualitative findings suggests a possible link between more frequent and diverse PA programming, and higher activity levels. However, given the descriptive nature of the analysis and uneven center sample sizes, these observations should be interpreted cautiously and viewed as hypothesis-generating rather than conclusive.

While all directors reported offering PA programs, the predominance of low-intensity, chair-based exercises reflects a risk-averse approach that may unintentionally reduce participants’ functional capacity. Similar findings in terms of PA program structure were reported by Rogerson and Emes (1), where ADC participants engaged in seated “gentle fitness classes,” however PA was not device-assessed in that study. Although ADC participants’ perspectives were not assessed in the present study, ADC participants from Rogerson and Emes (1) reported reducing functional decline and retaining independence as important factors of psychological resilience. While light PA has recently shown great promise for reducing mortality risk (28), evidence suggests that light-intensity activities are insufficient to improve physical performance or reduce fall risk (30). ADCs must reassess the effectiveness of their current programs, shifting from offering basic activity sessions to implementing structured, evidence-based interventions aligned with PA guidelines. In our study, directors primarily described light-intensity, chair-based or recreational activities (e.g., stretching, dance, seated movement), but did not report delivering standardized, manualized programs with demonstrated effectiveness (e.g., Otago, EnhanceFitness). This distinction highlights the gap between offering regular activity and implementing structured, evidence-based models designed to improve function and reduce falls.

Perhaps the most paradoxical finding relates to safety and liability concerns: all directors reported fear of falls as a primary limitation to the types of PA they implemented. In response to these concerns, there was an overemphasis on seated activities, despite evidence that prolonged sedentary behavior increases fall risk through muscle atrophy, poor balance, and reduced functional mobility (31). This cautious approach is understandable given that ADC participants demonstrated high fall risk, physical limitations, and increased mortality risk. Moreover, cognitive impairment was a common occurrence among participants and may have influenced both program participation and design decisions. When compared with other populations, our participants showed moderate physical impairments. For instance, older Brazilian adults in nursing homes had an average SPPB score of 6.14 units and a Timed Up and Go (TUG) score of 27.3 s (31), while our participants scored 8.79 units on the SPPB and 14.69 s on the TUG, indicating somewhat higher functioning despite high risks. However, when compared to community-dwelling older adults, our participants fared worse. Braun et al. (32) meta-analysis reported average SPPB scores ranging from 7.5 to 10.7 units and TUG scores between 8.2 and 14.6 s, suggesting that ADC participants have greater mobility impairments than the broader older adult population but less severe than those in institutionalized settings.

Prolonged reliance on seated activities can accelerate frailty and sarcopenia (33), creating a vicious cycle: fear of falls limits PA intensity, which in turn increases the likelihood of falls. Although liability concerns are valid, this overly cautious approach may prioritize institutional protection over participant well-being. There is a pressing need for balanced risk management strategies that promote safe yet effective PA interventions. Evidence-based fall prevention programs, such as the Otago Exercise Program, have demonstrated effectiveness even among high-risk populations through a focus on lower strength mobility, increasing functional mobility, and cognitive functioning (34). Additionally, the Fit & Strong Program incorporates low-mobility strength training and 30-min physical health education sessions, and has demonstrated improved functioning and quality of life (35). However, none of the centers in this study reported implementing such programs. Exploratory correlations from our sample further supported this need, showing that higher MVPA was associated with better physical function (SPPB, Chair Stand, and Gait). At the same time, MVPA was inversely associated with MMSE scores, a finding likely driven by the LUCID subsample, which combined lower cognitive scores with higher PA engagement. MVPA was also modestly lower among women, and income was positively related to cognitive scores, underscoring the intersecting role of gender and socioeconomic status in shaping both PA and cognitive health.

Staffing shortages and training were reported as the most significant barriers to implementing effective PA programs, underlining all themes. All directors cited this issue, particularly the challenge of providing personalized, higher-intensity interventions that require trained professionals. This finding aligns with prior research that documents the chronic underfunding of community-based senior programs, resulting in overburdened staff and limited capacity for specialized PA programming (6, 36, 37). PA programs led by trained professionals yield significantly improved functional outcomes (38, 39). Partnerships with community organizations, such as parks and recreation centers (40), can provide evidence-based PA programs, expand activity offerings, and support staff training. There is a need for mobilization from medical and healthcare professionals and encouragement of online programming to increase accessibility and feasibility (41, 42). Encouragingly, all directors expressed openness to collaborations and additional training, highlighting opportunities for capacity-building initiatives within ADCs.

While directors did not explicitly identify funding as a barrier, their responses implied that financial limitations affect program quality. Most ADCs in this study were nonprofit organizations, where existing funding may be insufficient to support specialized staff training, PA equipment, and the hiring of certified fitness professionals. This funding gap not only limits the scope of activities offered but also exacerbates liability concerns, as untrained staff may lack the skills to modify exercises safely for participants with complex health needs. Addressing these issues will require policy reforms and dedicated funding streams that prioritize PA programming and staff retention as an essential component of long-term care services (43, 44). Partnerships with universities, health departments, and community organizations could offer cost-effective solutions, such as staff certification programs and student-led PA sessions, that could be covered year-round without seasonal gaps, reducing staff burden and high turnover rates. Moreover, this disconnect between perceived and actual funding needs reflects a broader issue in health service delivery, where budgets prioritize basic care over proactive health promotion. Given the disproportionate impact on marginalized communities, targeted investments in PA programming are critical to promoting health equity among ADC participants.

Taken together, these barriers reflect not only immediate programmatic challenges but also broader structural limitations within ADCs. The reliance on volunteers and student interns, while expanding programming capacity in the short term, highlights gaps in organizational readiness and workforce sustainability. Similarly, funding constraints and liability concerns highlight systemic issues in the financing and regulation of community-based care. These structural barriers also align with domains of the RE-AIM framework, a globally recognized tool for evaluating not only the effectiveness of interventions but also their reach, adoption, implementation, and long-term maintenance across real-world settings (45). The RE-AIM framework offers a useful way to interpret these challenges, drawing attention to the organizational and contextual factors that shape whether programs are adopted, delivered with fidelity, and sustained over time. Viewing our results through this lens suggests that strengthening staff training and stabilizing funding may be just as critical as designing effective PA activities.

Beyond ADCs, similar dynamics, such as reliance on volunteers and limited staff training, also shape programming in other community-based settings like senior centers, where evidence-based programs such as Fit & Strong! and EnhanceFitness have demonstrated functional benefits but remain vulnerable to resource and staffing limitations (46, 47). However, findings may not generalize fully to assisted living or nursing home environments, which have distinct staffing models, regulatory contexts, and resident health profiles.

### 4.1 Strengths and limitations

This study has several strengths that enhance its validity and relevance. The multi-methods design, which combines device-assessed PA data with qualitative insights, provides a comprehensive view of PA patterns and program implementation within ADCs. Using ActivPAL devices minimized recall bias, providing objective measures of PA and sedentary behavior. Additionally, the diverse participant sample, comprising 70% Latino/Hispanic individuals and 76.9% with incomes below $25,000, enhances the generalizability of the findings to underserved populations often underrepresented in PA research. Data collected in real-world ADC environments further enhances ecological validity, offering practical insights into the challenges and facilitators of PA programming.

However, several limitations should be considered. Missing data was determined not at random, particularly from device-assessed PA measurements, which may have introduced bias. Although multiple imputation reduces potential bias, no sensitivity checks were conducted, and the model was limited to the available demographic and outcome variables. This may reduce the robustness of estimates; however, given the feasibility and descriptive focus of this study, we consider the approach appropriate for addressing missing data while preserving sample size. Moreover, low compliance with activity logs necessitated manual data cleaning, which could potentially impact data accuracy. It is important to note the average MMSE score, indicating mild cognitive impairment, which may have contributed to the low compliance rates of activity logs, loss of monitors, and overall missing PA data.

In addition, the number of participants varied substantially across ADCs (range *n* = 1 to *n* = 21). This uneven distribution limits the interpretability of center-level comparisons and may have influenced overall group estimates. Although the mean PA levels appeared high, the large standard deviation suggests substantial variability across centers. For this reason, our center-level findings should be interpreted as exploratory and with caution. Future studies with larger and more balanced samples may consider applying weighting, stratification, or statistical controls to reduce this bias.

Our exclusion criteria limited the sample to participants with moderate functioning. As a result, these findings may not generalize to more impaired populations, such as those with severe cognitive impairment, greater mobility limitations, or higher activity levels. Because PA levels are often lower in these more impaired subgroups, our results may underestimate the extent of low PA in the broader ADC population. Furthermore, the LUCID trial excluded participants reporting more than 150 min per week of structured PA, which may bias the combined sample toward lower baseline activity levels. However, it is important to note that LUCID participants in our dataset engaged in the highest levels of MVPA at baseline, suggesting meaningful variability across sites despite this criterion. Even so, the exclusion limits the generalizability of our findings to more active ADC populations.

Another limitation is that device-based data represent total daily PA and cannot be isolated to activity occurring specifically within ADCs. Attendance records were not available for all participants. As such, our findings reflect participants’ overall daily PA rather than activity directly attributable to ADC programming. However, capturing daily PA still provides important insight into the total activity levels and functional risks of ADC participants, highlighting the need for tailored programming in these settings.

Additionally, qualitative data may be subject to social desirability bias, as directors could have overstated program components. While the study included multiple ADCs, the relatively small sample size (*n* = 48) and the absence of interview data from one center director limit the generalizability of the qualitative findings. The missing qualitative data from this center may have introduced bias if it had unique practices or barriers not represented in the final analysis. The study also lacked participant perspectives, limiting insights into older adults’ personal experiences with PA programming, an essential factor for designing effective interventions. Regardless of limitations, our study adds insight to an under-studied population within ADCs, which may provide major public health implications.

### 4.2 Conclusion

Our findings highlight the urgent need for systemic changes to support PA promotion in ADCs. Studies should be conducted to examine how PA training for staff impacts the quality of PA programming and how these trainings translate to participant outcomes. Funding structures must be reevaluated to allocate resources specifically for PA programming, including hiring certified exercise professionals. Moreover, liability concerns should not reduce PA programs. Risk mitigation strategies, such as staff training in fall prevention, environmental modifications including supportive exercise equipment, and participant risk assessments, can help balance safety with the health benefits of more MVPA and structured programming. Future research should prioritize implementation pilots that evaluate structured, evidence-based programs (e.g., Otago, EnhanceFitness, Fit & Strong!) adapted for the cognitive and functional profiles of ADC participants. Incorporating frameworks such as RE-AIM can guide these pilots to assess not only effectiveness, but also reach, adoption, and sustainability within real-world settings. In addition, targeted staff training in exercise delivery and fall prevention will be essential for safe and scalable implementation. Community partnerships with local fitness centers, universities, and public health agencies may also expand PA opportunities year-round, including through student-led initiatives during the academic year, fostering healthier aging trajectories for ADC participants. Importantly, these findings should be interpreted as exploratory and hypothesis-generating, given the descriptive nature of the analyses and the uneven sample sizes across centers, which limit the generalizability of center-level comparisons.

## Data Availability

The datasets presented in this article are not readily available because the dataset supporting the findings of this study is not publicly available due to participant privacy concerns and restrictions imposed by the Institutional Review Board. De-identified data may be available from the corresponding author upon reasonable request and with appropriate institutional approvals. Requests to access the datasets should be directed to ysoto4@uic.edu.
